# Proteins, possibly human, found in World War II concentration camp artifact

**DOI:** 10.1038/s41598-022-16192-5

**Published:** 2022-07-20

**Authors:** Heyi Yang, Erin Butler, Samantha A. Monier, Donald Siegel

**Affiliations:** Office of Chief Medical Examiner, New York, NY 10016 USA

**Keywords:** Proteins, Mass spectrometry, Proteomic analysis

## Abstract

Museums displaying artifacts of the human struggle against oppression are often caught in their own internal struggle between presenting factual and unbiased descriptions of their collections, or relying on testament of survivors. Often this quandary is resolved in favor of what can be verified, not what is remembered. However, with improving instrumentation, methods and informatic approaches, science can help uncover evidence able to reconcile memory and facts. Following World War II, thousands of small, cement-like disks with numbers impressed on one side were found at concentration camps throughout Europe. Survivors claimed these disks were made of human cremains; museums erred on the side of caution—without documentation of the claims, was it justifiable to present them as fact? The ability to detect species relevant biological material in these disks could help resolve this question. Proteomic mass spectrometry of five disks revealed all contained proteins, including collagens and hemoglobins, suggesting they were made, at least in part, of animal remains. A new protein/informatics approach to species identification showed that while human was not always identified as the top contributor, human was the most likely explanation for one disk. To our knowledge, this is the first demonstration of protein recovery from cremains. Data are available via ProteomeXchange with identifier PXD035267.

## Introduction

**Figure 1:**
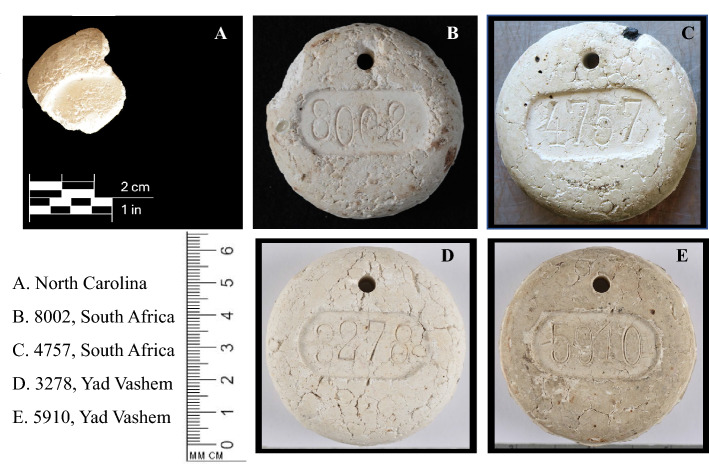
Images of disks analyzed by the NYC OCME.

On a visit to liberated Dachau concentration camp in 1945 a member of the United States Army was given a small, cement-like, disk-shaped object by a former prisoner; the disk (only a fragment remains) was said to be made of human cremains (Fig. 1A, Extended Data Fig. 1)^1^. Around the same time, a South African soldier also visited Dachau and was given a similar disk with a hole near its top and a number imprinted on the disk’s surface during its casting (Fig. 1B, Extended Data Fig. 1)—he was also told by a surviving prisoner that the disk was made from human cremains^2^. Similar looking disks may be found on display at Holocaust museums around the world (Fig. 1C–E and Extended Data Fig. 2A–E). The composition and function of these disks have been a matter of debate. Archivists^2^, without documentation about disk production and function in concentration camps during WWII can only state that in Germany, prior to the war, similar looking cement-like or fireclay disks with numbers on them were used in civil cremation^2^ (not unlike today, they were placed with a decedent in a crematory retort to ensure that an individual’s cremains were not misidentified). Survivors and later owners of the disks claimed that in the concentration camps the disks were used as “coat checks”, i.e. given to arriving prisoners in exchange for their clothes or baggage (as a ruse to prevent panic before execution, Extended Data Fig. 2D, E) or they were dipped in fat and given as “soap” in a similar ruse (Extended Data Fig. 2A, B). Knowledge of the composition of these disks, particularly if they are composed of biological material, may help evaluate the testimony of survivors with respect to the use of human cremains in disk production, as well as their subsequent function. The challenge is to extract and detect species specific biological material following cremation. A proteomic high performance liquid chromatography/tandem mass spectrometry approach (employing a new informatics method for species identification^3^) was used to evaluate the disks as proteins: encode genetic information, are more stable than DNA to environmental insult, and have been found further back in the geological record (> 1,000,000 years)^4–7^. Proteins and DNA have previously been detected in burned bone^8–10^ but not, to our knowledge, in crematorium retort cremains.

### Proteins in species identification

Species identification by proteomic mass spectrometry is typically performed by identifying species specific peptides in a sample^11–13^. However, protein-based species identification can be much more difficult when only a small number of proteins are identifiable—which inevitably limits the pool of species informative peptides. Limited protein detection can result from sample degradation, or because a single protein dominates the samples and consequently limits detection of other less abundant but species informative proteins—i.e. proteins outside the dynamic range of detection. Bone is a classic example. Collagen is by far the predominant protein detected in bone^14,15^, and even in a known single source bone sample, collagen spectra often match to multiple species when searched against a large vertebrate database (as would be necessary with an unknown sample) with no clear “winner” species identifiable^3^. Incorrect collagen spectra identifications, and consequently incorrect species identifications, can result from the limitations of tandem mass spectrometry (e.g. incomplete peptide ion fragmentation, especially in fragment ions with species specific amino acid position inversions), posttranslational modifications, algorithm interpretation errors, or the inability to distinguish isobaric amino acids (e.g. leucine and isoleucine)^3^. Species identification is a particularly difficult problem with collagens. For not only are they the predominant protein identified in bone^14^—i.e. limiting the detection of other less abundant proteins (see above), they are highly conserved through evolution^15^, limiting, to some extent, their use for species identification. Additionally, because bone collagens require significant levels of proline hydroxylation for biological activity^14,16^, the common collagen dipeptide sequence hydroxyproline-alanine is frequently interpreted as the isobaric sequence proline-serine—an additional cause of species misidentification^3^. Two methods for improving species identification from bone are: (1) selecting the “winner” species based on the greatest number of species-assigned spectra (as opposed to peptides), which reduces, but does not eliminate, multiple species identifications^3^, and (2) reducing false positives by limiting database search space to those species from which the sample is likely to have come^3,17^. For species identification of the disks, the spectra species-assignment method^3^ was used. Database search space was limited to all European mammals and common domestic animals^18^; additional related entries were added to increase the likelihood of a match (Methods, Supplementary Table 1). After species identification by spectra, subsequent analyses were performed on identified species peptides.

## Results

A total of 1183 protein accession numbers were found with high quality peptide spectral matches (PSMs) (Scaffold probability ≥ 98%) in all five disks, spanning 159 species and 7 taxonomic orders. (These proteins are non-redundant within species, but may be shared between species, see Supplementary Table 2.) To avoid contaminant proteins that may have been introduced to the surface of the disks over intervening decades (e.g. those found in skin or saliva), samples were analyzed only for collagens 1A1 and 1A2 (COL1A1, COL1A2), and hemoglobins alpha and beta (HBA, HBB), two of the most abundant proteins in the body that are not typically found in skin or saliva.

A total of 106 distinct collagen and 10 hemoglobin peptides were identified (Scaffold probability score ≥ 98%)  (Supplementary Table 3 and 4). All were manually searched against NCBI’s Basic Local Alignment Search Tool (BLAST) to confirm that no sequences matched to other organisms—e.g. invertebrates or microorganisms. Data from samples extracted multiple times were merged and searched against the custom database as a single file (Materials and Methods).

**Figure 2:**
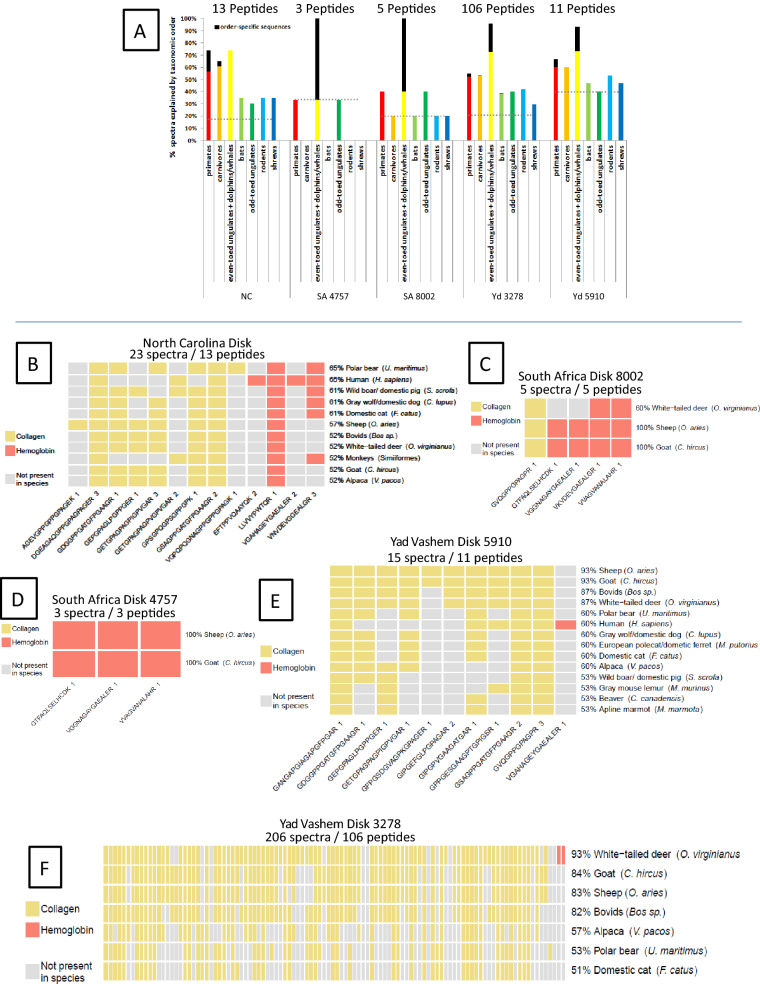
Relative ranking of species and order by peptides identified in five WWII disks. (**A**) ranks the disks by orders. The percent of spectra below the dotted lines represent peptides shared between all orders in a given disk. (**B**)–(**F**) X-axes identify all peptides found in a particular disk as well as the number of times that peptide was independently detected. (Sequences for (**F**) may be found in Supplementary Table 3) Y-axes indicates possible species assignments using different combinations of the identified peptides. Percentages reflect the number of peptides used for a species assignment as a total of all peptides identified (only species that explained > 50% of the data are shown). Only in the North Carolina disk (**B**) is human the most likely species interpretation (65% of peptide, tied with polar bear*). The only other disk with human specific peptides is Yad Vashem disk 5910 where human represents the third largest number of peptides (60%, again tied with polar bear), while sheep/goat (93%) and cow/deer (87%) are higher. *Identification of non-European wildlife, e.g. polar bear, Virginia white-tailed deer, alpaca, etc. are likely due to limitations of databases for the European species of these genera. For example, both polar bears (*Ursus maritimus*) and Eurasian brown bears (*Ursus arctos arctos*) are of the same genus.

Four of the disks yielded similar numbers of peptides (Fig. 2B–E), with an average of eight and a range of 3–13. The fifth disk, #3278 yielded 106 peptides (Fig. 2F). The 116 collagen and hemoglobin peptides identified from all disks matched to 109 species representing seven mammalian orders (Extended Data Fig. 3A) with 29.3% of peptides unique to a single order, and 17.2% shared between all seven orders (Extended Data Fig. 3B, Supplementary Table 3). Figure 2A shows that four of the five disks had peptides from all seven orders. While some of these peptides are shared between orders (i.e. are not order specific-areas below dotted line Fig. 2A) and therefore might be expected in a disk composed of bone from a single species, some peptides are not shared between orders (area above dotted line Fig. 2A), and suggests that those disks could be composed of bone from more than one order. (In an analogous way, species specific peptides from within a single order can also be found within the same disks, e.g. sheep specific and cow specific peptides from the order Cetartiodactyla in disk 5910 (Fig. 2E)). These data may suggest that the disks are composed of more than one species—e.g. disks NC, 3278 and 5910 all have primate specific and/or Cetartiodactyla or Carnivora specific peptides. However, a similar phenomenon—i.e. identification of multiple distinct orders or species-specific peptides in a single source sample—has also been observed in bone^3^, due to the reasons discussed above (large database search size, mass spectrometer limitations, post-translational modifications single amino acid isobaric changes, and algorithm interpretation errors).

One method for assigning a single species to a sample (e.g. a single source bone) with high quality PSMs that include multiple species is to choose the winner species as that species which has the greatest number of assigned spectra—both unique to that species and shared with other species^3^. Figure 2B–F shows the peptides identified in each disk (x-axis) and the species (y-axis) which use the greatest combination of these peptides (ranked by percentage highest to lowest). Thus, for example, in the North Carolina disk fragment both human and bear account for 65% of all spectra (Fig. 2B, Supplementary Table 5). In disk 5910 (Fig. 2E, Supplementary Table 5), sheep and goat tie at 93% of accounted spectra while human accounts for 60% of assigned spectra. In disk 3278 (Fig. 2F, Supplementary Table 5), with the most identified peptides (106), deer account for 93% of spectra, while goat, sheep and cow account for 83% of assigned spectra—all these species are from the order Cetartiodactyla. Using this method, which assumes only one species is present in a disk, only the North Carolina disk would be considered likely composed of human cremains (cremation of bears at Dachau seems unlikely), even though disks 3278 and 5910 have human specific peptides.

**Figure 3:**
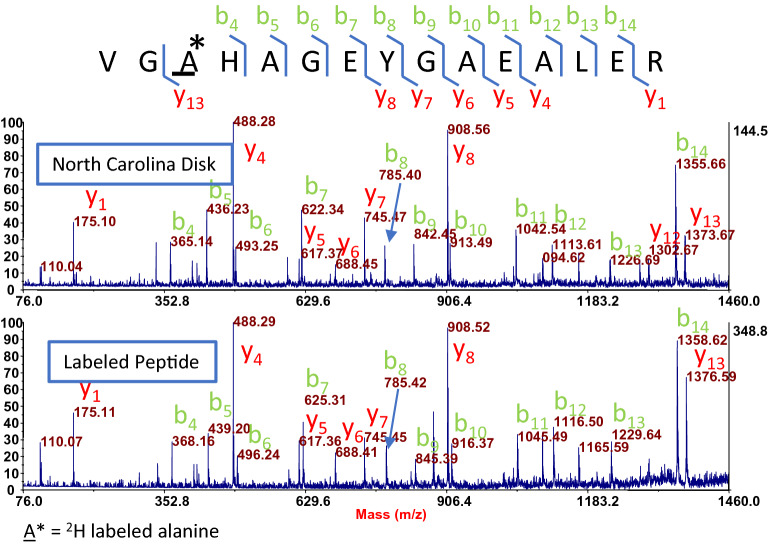
Comparison of North Carolina hemoglobin peptide VGAHAGEYGAEALER spectrum with ^2^H labeled synthetic peptide. Spectral comparison between human hemoglobin peptide VGAHAGEYGAEALER (shared with *Pan paniscus* (bonobo) a close primate relative of humans) identified in the North Carolina disk, and its synthetic counterpart with matching b and y ions.

One of the most reliable MS/MS methods for confirming peptide identity is comparing a sample spectrum to the spectrum of a synthetic peptide to which it is believed to match. To evaluate the two primate hemoglobin peptides found in the North Carolina disk, their spectra were compared to spectra from isotopically labelled synthetic peptides. Figure 3 shows a spectral comparison between human hemoglobin peptide VGAHAGEYGAEALER and its synthetic counterpart with matching b and y ions. The spectra match is nearly identical. A similar match with synthetic human hemoglobin peptide VNVDEVGGEALGR is also seen in the North Carolina disk extracts (Extended Data Fig. 4).

## Discussion

Historians are reluctant to attribute horrific crimes to genocidal regimes on the basis of survivor or eyewitness testimony without documentary evidence, as even a single false claim can become a tool to refute actual crimes by genocide deniers^2,19^. This level of required certainty can place historian and archivists in the uncomfortable position of appearing to question survivor testimony—a situation explored by Jeff Cohen in *The Soap Myth* ^20^, a play about the controversial claims that Nazis rendered the fat of Jews into soap^19^. The use of cremains in the manufacture of the disks describe herein is another controversy between the demands of documentation by archivists and survivor testimony. Advances in scientific techniques and methods have the potential to address and, perhaps in this case, reconcile these differences.

Species identification of cremains by proteomic mass spectrometry is complex as little biological material remains after burning^8^, and in what does remain, potential amino acid losses or sidechain modifications^21–23^ could change the mass and consequently the species identification. Interpretation is further complicated by the inevitable false positives that result from searching a small number of spectra against a large database of vertebrate protein sequences. However, the data presented here clearly demonstrate that biological material is contained within what were believed to be cement disks^2^, and that material is consistent with bone. Collagen, the most abundant protein in bone^14,16^, has increased protection from burning due to the large amounts of inorganic calcium that make up the bulk (~ 65%) of bone mass^24–26^. Marrow, a rich source of hemoglobin, and the second most abundant protein in the body, may also have some protection from the intense heat that consumes exposed soft tissues. (It should be noted that while COL1A1 is expressed in the skin’s inner dermal layer, it is not commonly found in the epidermis (the outermost layer of skin)^27^. Indeed, COL1A1 and COL1A2 have not been identified in the transcriptomes or proteomes of the two most abundant cell types found in the epidermis, keratinocytes, which constitute the bulk of the epidermis, and corneocytes, which derive from keratinocytes^28–31^ and are shed, and deposited during touching. Collagen types IV, VII and 17A1 are found in the epidermis, but it is the proteins keratin and filaggrin that comprise 80–90% of the mass of the epidermis, and protein loricrin contributes 70–85% to the mass of the cornified cell envelope^27^. Consequently, because COL1A1 and COL1A2 are not found in keratinocytes and corneocytes proteomes, we believe that their detection on the surface of Yad Vashem disk numbers 3278 and 5910 are not likely to be a consequence of touch contamination. South Africa disks 3278 and 5910, as well as the North Carolina disk fragment (Extended Data Fig. 1) were sampled from their interiors).

Detection of the two most abundant body proteins in these disks strongly suggests that cremains were used in their manufacture. This leads to a fundamental question: Why might bone cremains be used in making these disks? One possible answer is that in Germany during World War II, a time of scarce resources, cremains were used as a substitute source of aggregate and/or, because its lime content (calcium oxide) was believed to be a substitute, or partial substitute, for cement^32–34^. This in turn leads to another question: What important function did these disks have that they needed to be continually produced during a massive war effort? Dachau, the first Nazi concentration camp, opened in 1933 to hold political prisoners. Those who died in the camp could be cremated and their ashes returned to their families upon payment of costs and shipping^35,36^. A photograph of liberated Dachau^37^ shows a storage room filled with cremation urns. A similar photograph of liberated Natzweiler-Struthof concentration camp (Bas-Rhin, France) ^38^, shows storage of cremation urns and what appear to be disks similar to the ones described herein. As the war progressed large numbers of prisoners were brought to Dachau as laborers and died. Their bodies were not cremated to be returned to families, but to hide evidence of mass killing^39^—yet still the markers were produced. Why? Evidence from the Yad Vashem and the Majdanek State Museum collections of disks suggests that the disks were used as “coat checks” for belongings of prisoners (Extended Data Fig. 2D, E), or covered in fat to be used as “soap” before prisoners were led into gas chambers (Extended Data Fig. 2A, B). Similar ruses (e.g. the numbering clothes hooks in the anterooms to gas chambers) were used as a way to prevent panic at Auschwitz^40^. Evidence that the disks may have been covered in fat (in addition to survivor reports) is from the archivist at Yad Vashem who stated that some of the disks are darker than others and these appear greasy (personal communication). If the disks were used as “coat checks” at Dachau, it would explain why they were continually produced, even to the end of the war—they were repurposed from their original function of identifying individuals in civil cremation to become a ruse to prevent panic as increasing numbers of prisoners arrived.

The identification of peptides in what appears to be cement disks was unexpected. The identification of multiple species peptides is not surprising as it is a common finding even in single source bone samples^3^. That there are fewer peptides detected in cremains than unburnt bone makes determining whether these disks are from a single species (including human) more difficult. Further, commingling of human remains may have been inherent to the cremation process at Dachau as it was at Auschwitz^40^, and the presence of non-human collagens, if indeed present, may be the result of the occasional burning of the remains of domestic food animals (a breeding station at Dachau housed more than 4000 animals in 1944^41^) or local wildlife in the same retort used for human bodies, although we do not know of any such evidence.

Museum archivists are responsible for accurately representing their collections to the public without prejudice and based on the best available evidence^2,19^. Previously the only evidence that these disks were made from cremains came from survivors. The fact that the five disks tested here had collagen and hemoglobin in them seems to confirm survivor testimony. Whether these disks were made with human or Cetartiodactyla cremains or combination of them is difficult to say. However, these data give credence to survivor statements that the disks were made from cremains.

## Materials and Methods

### Materials

Reagent/small equipment abbreviations and sourcing

- Acetone Chromasolv Plus for HPLC (Sigma, St. Louis, MO)
- Acetonitrile (ACN) HPLC plus (Sigma, St. Louis, MO)
- Acetonitrile 0.1% Trifluoroacetic acid (ACN, TFA) HPLC grade, (Fisher, Fairlawn, NJ)
- Alpha-cyano-4-hydroxycinnamic acid (Bruker, Sigma, St. Louis, MO)
- Ammonium bicarbonate (ABC) (Fluka, Sigma, St. Louis, MO)
- Bradford Reagent (Sigma, St. Louis, MO)
- C18 tips (Pierce, Rockford IL)
- Dithiothreitol (DTT) BioRad (Hercules, CA)
- Hydrochloric acid (HCl) (Sigma, St. Louis MO)
- Iodoacetamide (IAA) (Sigma, St. Louis, MO)
- Tris(2-carboxyethyl) phosphine hydrochloride (TCEP) pH pre-adjusted with ammonium hydroxide (Sigma, St. Louis MO)
- Trifluoroacetic acid (TFA), Optima LC/MS grade (Fisher, Fairlawn NJ)
- Trypsin Gold, Mass Spectrometry Grade (Promega, Madison WI)
- Urea (Sigma, St. Louis, MO).

### Cremation disks

(See Extended Data Table 1 for provenance.) Three of the five disk were sent to the NYC OCME for sampling. These disks were sampled from their interiors (see below). The remaining two disks were sampled from their surface at the Yad Vashem Holocaust Museum and the samples sent to the NYC OCME. The number of times each disk was sampled, and the weight of each sample are described below.

- North carolina disk: The NC disk fragment (Fig. 1A, Extended Data Fig. 1) was sent to the NYCOCME for sampling. The fragment was brittle with small pieces occasionally breaking off. One of the pieces that naturally broke away from the disk weighed 61 mg. A second piece, taken from inside the disk at the NYC OCME (i.e. not exposed to the environment or touched) weighed 75 mg. Both were analyzed and showed similar results with respect to peptide sequences and the number of collagen and blood peptides identified.
- Yad Vashem Disk #3278: A 56.9 mg sample of this disk was taken from the disk’s surfaces at the Yad Vashem Holocaust Museum and sent to the NYC OCME for analysis.
- Yad Vashem Disk #5910: A 50 mg sample of this disk was taken from the disk’s surfaces at the Yad Vashem Holocaust Museum and sent to the NYC OCME for analysis.
- South Africa Disks #4757: This disk was sent to the NYC OCME by The Cape Town Holocaust & Genocide Centre, SA. A portion of the disk’s surface was removed and ~ 200 mg of material recovered from its interior. This material was divided in two (97 and 103 mg) and each sample extracted as describe below.
- South Africa Disks #8002: A portion of the disk’s surface was removed and ~ 252 mg of material recovered from its interior. This material was divided in two (124.4 and 127.6 mg) and each sample extracted as describe below.

### Protein extraction

Disk samples were demineralized in 1.2 M HCl, acid supernatants discarded and resulting pellets extracted first with 8 M urea and then reextracted with 0.6 M HCl. Both urea and 0.6 M HCl extracts were processed by LC/MS. This method was used on all disks except the North Carolina disk, which was only extracted using 8 M urea, details below.

Disk samples were demineralized in ten volumes (wt/wt) 1.2 M HCl overnight at 4 °C. Following demineralization, samples were centrifuged, supernatants discarded, and pellets washed three times with ~ 200 μl of water/wash until pH approached neutrality. To extract proteins from the pellet, 45 μl of 8 M urea (in 50 mM ABC) were added to each sample and the pellets incubated for 72 h at 4 °C. (For NC disk samples, extraction was at room temperature for 30 min.) Samples were again centrifuged, and in-solution trypsin digestion performed on supernatants (see digestion method below). Pellets (except of the NC disk) were reextracted, this time with 50 μl 0.6 HCl with shaking at 70 °C for 1 h. Samples were again centrifuged, and this time pellets discarded. To the 0.6 M HCl supernatants five volumes of cold acetone (-20 oC, v/v) were added. Proteins from samples were left to precipitate overnight at − 20 °C and again centrifuged. Supernatants were discarded and precipitates allowed to dry at room temperature. Precipitates were solubilized with 50 μl of 8 M urea (in 50 mM ABC) for 30 min at room temperature. In-solution trypsin digestion was performed on supernatants and peptides analyzed by LC/MS (see LC/MS methods below).

1. Analysis of the NC disk’s 1.2 M HCl demineralization solution identified only one peptide. Consequently, for subsequent disks, the 1.2 M HCl demineralization solution was discarded, and a second pellet extraction using 0.6 M HCl followed by acetone precipitation was added.
2. Bradford protein assays of the 8 M urea and 0.6 M HCl/acetone precipitated samples were negative. LC/MS was performed on one third to one half of digested sample.

### Trypsin digestion

All samples were reduced with 5 mM TCEP (in 50 mM ABC) for one hour at 37 °C, alkylated with 15 mM IAA (in water) for 30 min in the dark at room temperature, and quenched with 10 mM DTT (in 50 mM ABC) for 15 min at room temperature. Samples were digested with 250 ng trypsin on a shaking incubator at 37 °C overnight. Samples containing high concentrations of urea were diluted to 2 M urea with 50 mM ABC before trypsin digestion.

### Reverse phase high-performance liquid chromatography

All samples were brought to 0.1% TFA and then processed through 100 μl C18 tips as follows. Tips were treated as mini-C18 columns; samples were pipetted onto the resin and then centrifuged for 30 s at 100 g. Flow-throughs were reapplied four additional times. Samples were eluted with 100 μl 50% ACN using the same centrifugation method. Eluates were reapplied three additional times and dried to completion in a SpeedVac. Samples were resuspended in 20 μl 2% ACN, 0.1% TFA and subsequently centrifuged for at 18,000 g 30 min at 4 °C to pellet debris.

High-performance liquid chromatography (HPLC) was performed on a Dionex UltiMate 3000 instrument (Dionex, Sunnyvale, CA). Tryptic peptides were desalted using an inline C18 trap column (300 μm × 5 mm, 100 Å 5 μm particle size, Dionex) and separated on an analytical PepMap100 RP C18 column (75 μm × 150 mm; 100 Å 3 μm particle size, Dionex) with an ACN elution gradient of 5–40% containing 0.1% TFA at flow rate of 300 nl/min run with inline mixing of alpha-cyano-4-hydroxycinnamic acid matrix (7 mg/ml in 75% ACN, 25% water, 0.1% TFA). Samples were run on 40 min linear gradient collecting 96 aliquots on a MALDI plate. Prior to running samples, blank runs of 2% ACN 0.1% TFA were inspected by inline UV to ensure the columns were free of carry over. For the NC and South Africa disk #8002, blanks were run on the mass spectrometer.

### Tandem mass spectrometry

MS data were acquired on a 4800 MALDI TOF/TOF (Sciex, Framingham, MA) at a laser repetition rate of 200 Hz with 1500 laser shots/spectrum (50 laser shots/sub-spectrum). MS/MS data were acquired at 200 Hz in 1 kV MS/MS mode with 3500 laser shots/spectrum (50 laser shots/sub-spectrum) with the following TOF/TOF Series Explorer Stop Conditions: maximum shots per spectrum 3500, minimum shots per spectrum1000, number of MS/MS fragments 8, S/N of each fragment 75. The top 30 strongest peaks were selected for MS/MS.

### Data analysis

Database size reduction was performed as follows. First, a broad initial search of the disk data was performed against the National Center for Biotechnology Information’s (NCBI) non-redundant vertebrate database. This search revealed samples to be mammalian, with few peptides matching other vertebrate classes. To refine the search space, a custom database was created of all available European mammals compiled in the International Union for Conservation of Nature’s European Mammal Assessment[18], plus common domestic animals (Supplementary Table 1). Species included those found to inhabit any of the 25 countries that were part of the European Union at the time of the IUCN assessment. Domestic animals included cow, goat, sheep, llama, alpaca, house cat, donkey and horse. (Note: Some domestic animals share scientific names with their wild counterparts and so were already on the list, e.g. dog/wolf, pig/boar.) However, because not all the species had published proteomes in NCBI, the database was broadened to include the protein sequences of any species that shares a genus with any of the targeted European/domestic mammals. To aid in detection of known human polymorphisms, peptide sequence variants from the National Heart, Lung and Blood Institute Grand Opportunity GO Exome Sequencing Project were included in the custom database (May 2018). All available primates were included in the search in order to capture potential human peptides that might be isobaric to non-human primate peptides (e.g. I > L). (All primate identified peptides were also identified as human, however). Data were searched against NCBI non-redundant vertebrate database (April 2018) and our own database by Mascot (Matrix Science, Boston, MA) and ProteinPilot (Sciex, Framingham, MA). The parameters for Mascot searching were as follows; protein modifications: carbamidomethylation of C (fixed modification), deamination of Q and N, oxidation of M and P (variable modification); mass values: monoisotopic; protein mass: unrestricted; peptide mass tolerance: ± 250 ppm; fragment mass tolerance: ± 0.35 Da; max missed cleavages: 2. The parameters for ProteinPilot searching; sample type: identification; C alkylation: iodoacetamide; digestion: trypsin; instrument: 4800 MALDI TOF/TOF; special factors: none; species: none; search effort: thorough.

Search results from both Mascot and ProteinPilot were imported to Scaffold (Proteome Software, Portland, OR) and reprocessed by Scaffold. Trypsin and keratins were assumed to be contaminants and removed. Only spectra with a Scaffold probability > 98% were considered.

## Supplementary Information

Supplementary Information 1.

Supplementary Information 2.

Supplementary Information 3.

Supplementary Information 4.

Supplementary Information 5.

Supplementary Information 6.

Supplementary Information 7.

Supplementary Information 8.

Supplementary Information 9.

Supplementary Information 10.

## Data Availability

The mass spectrometry proteomics data have been deposited to the ProteomeXchange Consortium via the PRIDE^42^ partner repository with the dataset identifier PXD035267 and 10.6019/PXD035267.
