# Supplementary material for: Proteins, possibly human, found in World War II concentration camp artifact
Source: Sci Rep. 2022 Jul 20;12:12369. doi: 10.1038/s41598-022-16192-5 (PMC9300652; doi:10.1038/s41598-022-16192-5)
Supplement: Supplementary file 1 — Supplementary Information 1. [file 41598_2022_16192_MOESM1_ESM.pdf]

Extended Data Figure 1

Comparison of North Carolina Disk Fragment with South Africa Holocaust Museum Disk 8002

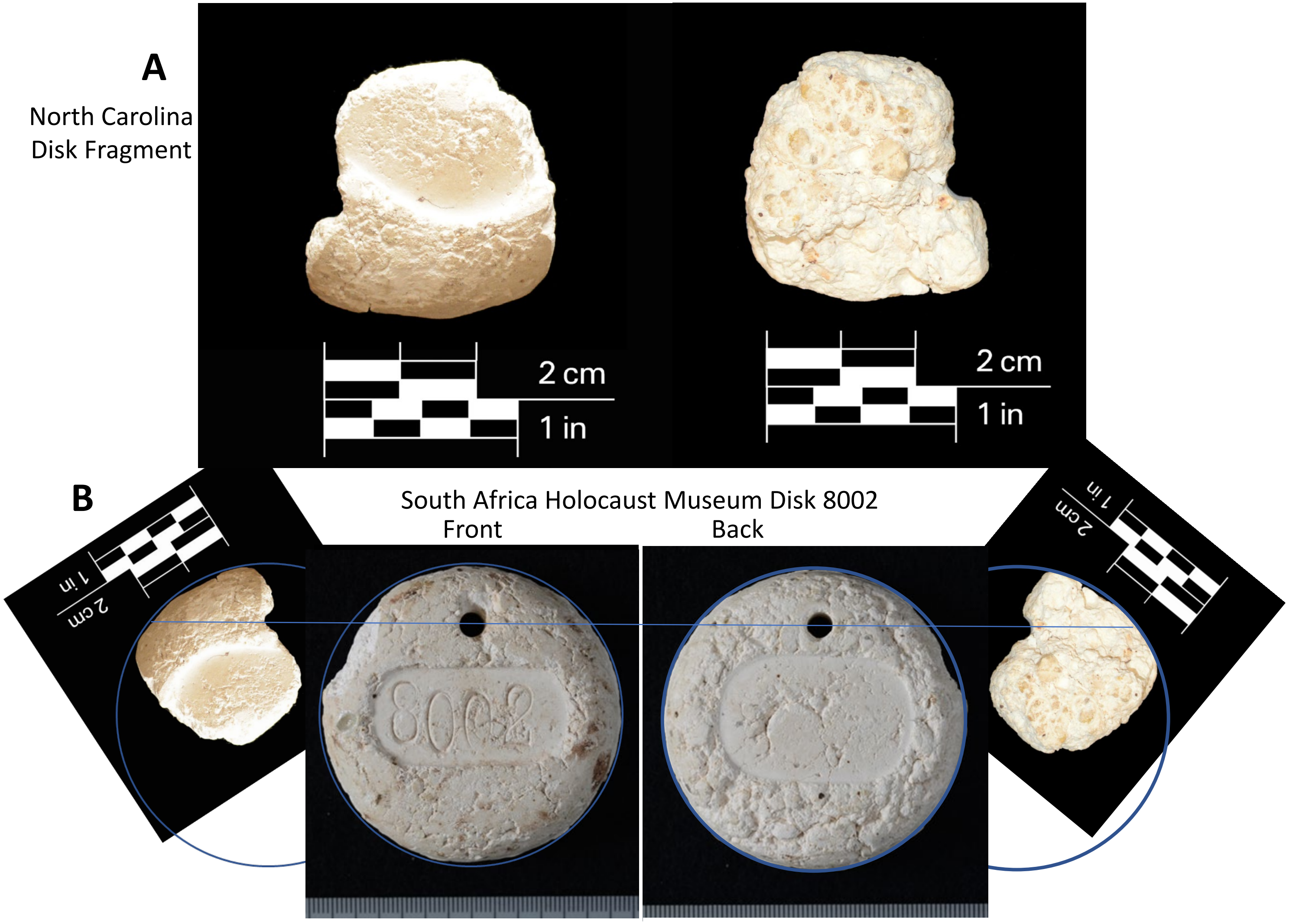

Legend – A comparison of the North Carolina disk fragment with South Africa Holocaust Museum Disk 8002 shows that the molded indentation on the fragment surface (A, left side) is similar in size and appearance to Disk 8002, that the circumference of the fragment would be consistent with the full disk (circumference of disks is ~63 mm), and that the “smooth bore” hole (seen most easily on the back of the fragment, A, right side) appears to have been molded or drilled into what would have been an original disk. Blue circles around Disk 8002 and fragment in Panel B are equal in diameter. Blue line courses through the center of the holes.
