# Supplementary material for: Proteins, possibly human, found in World War II concentration camp artifact
Source: Sci Rep. 2022 Jul 20;12:12369. doi: 10.1038/s41598-022-16192-5 (PMC9300652; doi:10.1038/s41598-022-16192-5)
Supplement: Supplementary file 2 — Supplementary Information 2. [file 41598_2022_16192_MOESM2_ESM.pdf]

Extended Data Figure 2

Photographs of disks from Yad Vashem's and Majdanek State Museum's Artifacts Collections not tested by NYC OCME

Details of "Cement" Tags in Yad Vashem's Artifacts Collection*

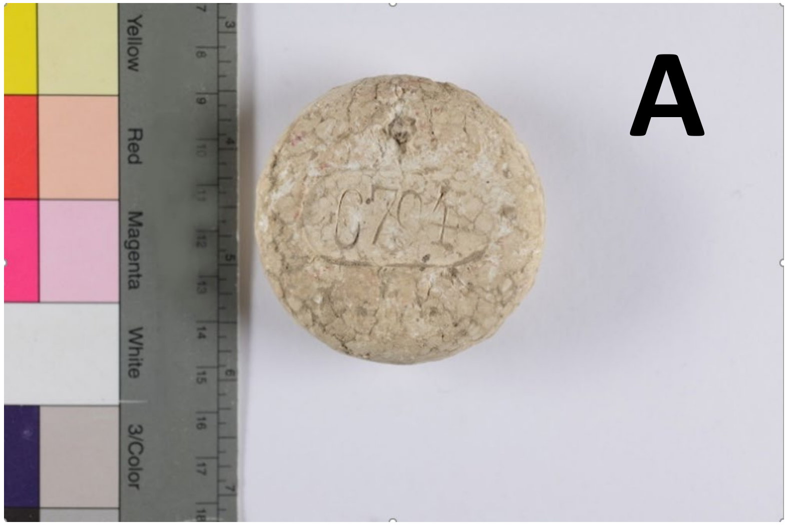

YV #1321 - cement tag imprinted with number **6794**
Diameter: 6.3 cm. Thickness 2 cm.
Provenance: Details regarding provenance: On a visit to the Auschwitz camp site in 1955 it was found by a Romanian journalist who kept it until 1972, when he donated it to Yad Vashem. He stated that at the time he was told by Poles that it was covered in fat and used as soap that was given to those prior to being sent to the gas chambers.

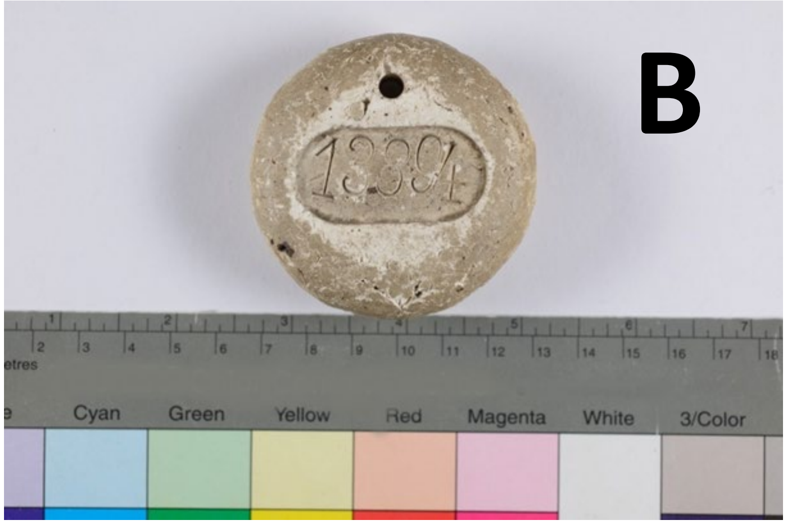

YV #1522 - cement tag imprinted with number **13894**.
Diameter: 6.2 cm. Thickness 1.8 cm.
Provenance: No details regarding provenance: donated to Yad Vashem in 1977 by a resident of Jerusalem as "a bar of soap". No further information was provided. Disk has five numbers imprinted on it.

All disks have an indentation on their reverse side identical to the indentation on their front but without a number, except for E. Disks YV#1321 (**A**), YV#1522 (**B**), YV#2706 (**C**) and MMP-I-12136 (**E**) are darker than disk YV#11389 (**D**).

* Descriptions from Sara Shor, Artifacts Collection, Yad Vashem Museum, Israel

Extended Data Figure 2

Photographs of disks from Yad Vashem's and Majdanek State Museum's Artifacts Collections not tested by NYC OCME

Details of "Cement" Tags in Yad Vashem's Artifacts Collection*

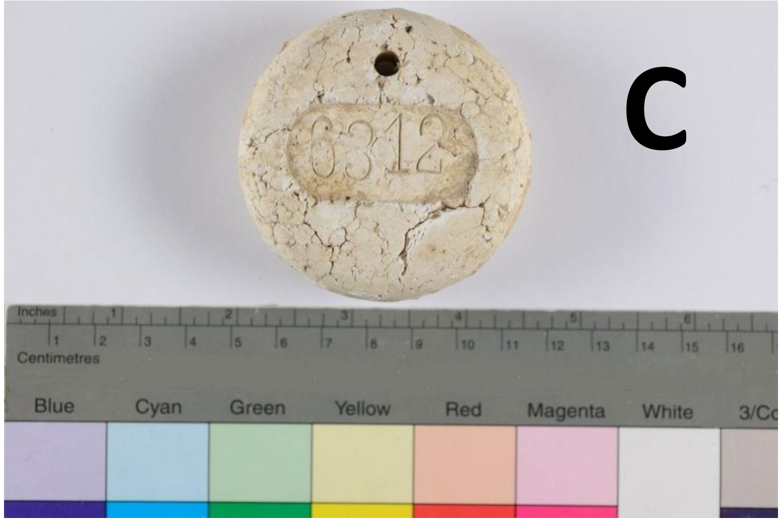

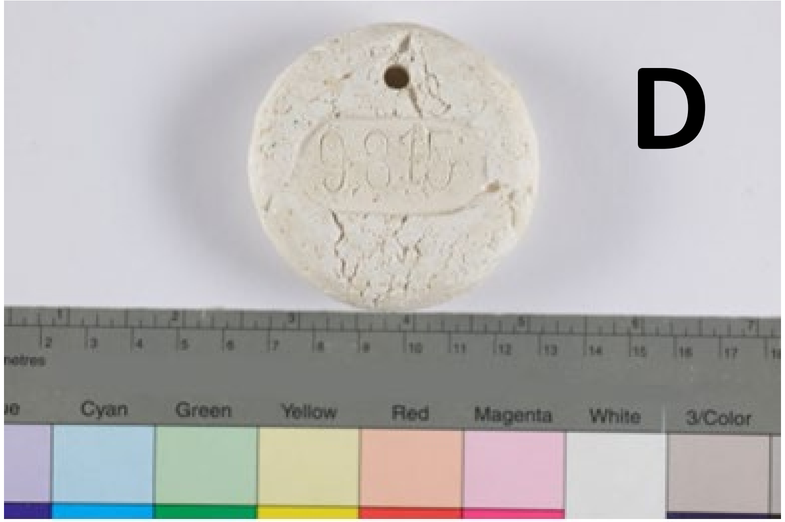

YV #2706 - cement tag imprinted with number **6312**
Diameter: Diameter: 6.3 cm. Thickness 2 cm
Provenance: Found by a resident of Petach Tikva in Auschwitz in 1948. Donated to Yad Vashem in 1999.

YV #11389 - cement tag imprinted with number **9815**
Diameter: 6.3 cm. Thickness 1.8 cm.
Provenance: Found in a flea market in Illinois, US who was told by the seller that it was a tag for left belongings given to Jews who deposited their belongings before entering the gas chambers. Donated to Yad Vashem in 2011.

All disks have an indentation on their reverse side identical to the indentation on their front but without a number, except for E. Disks YV#1321 (**A**), YV#1522 (**B**), YV#2706 (**C**) and MMP-I-12136 (**E**) are darker than disk YV#11389 (**D**).

* Descriptions from Sara Shor, Artifacts Collection, Yad Vashem Museum, Israel

Extended Data Figure 2

Photographs of disks from Yad Vashem's and Majdanek State Museum's Artifacts Collections not tested by NYC OCME

Details of "Cement" Tags in Belzec - Majdanek State Museum Artifacts Collection‡

E Front

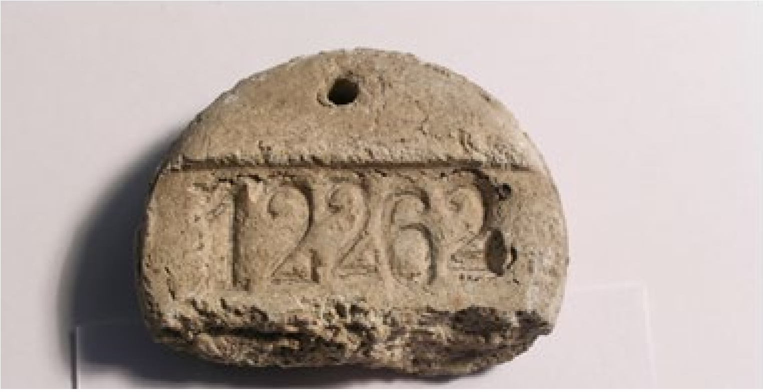

E Back*

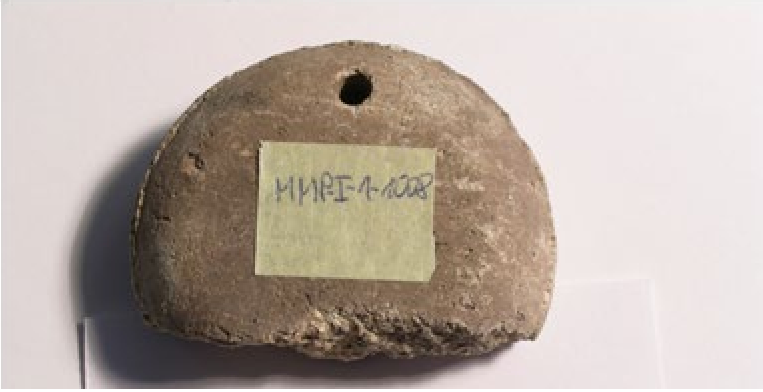

"Found during excavations at Belzec. We think that Germans used them in the Belzec death camp as a kind of receipt for valuable items which people brought with them (it was a part of procedure before sending people to gas chambers)."

All disks have an indentation on their reverse side identical to the indentation on their front but without a number, except for E. Disks YV#1321 (**A**), YV#1522 (**B**), YV#2706 (**C**) and PMM-B-2705 (**E**) are darker than disk YV#11389 (**D**).

‡ Descriptions from Tomasz Hanejko - Head of MMPB, Museum – Memorial Site in Bełżec, ul. Ofiar obozu 4, 22-670, Bełżec, Poland

* **N.B.** Disk E identification number MMP-I-1-1008 (in photograph) has been changed to PMM-B-2705.
