# Supplementary material for: Proteins, possibly human, found in World War II concentration camp artifact
Source: Sci Rep. 2022 Jul 20;12:12369. doi: 10.1038/s41598-022-16192-5 (PMC9300652; doi:10.1038/s41598-022-16192-5)
Supplement: Supplementary file 3 — Supplementary Information 3. [file 41598_2022_16192_MOESM3_ESM.pdf]

Extended Data Figures 3 A & B
Analysis of Unique and Shared Peptides by Taxonomic Order

3A Number of shared and unique peptides in each order

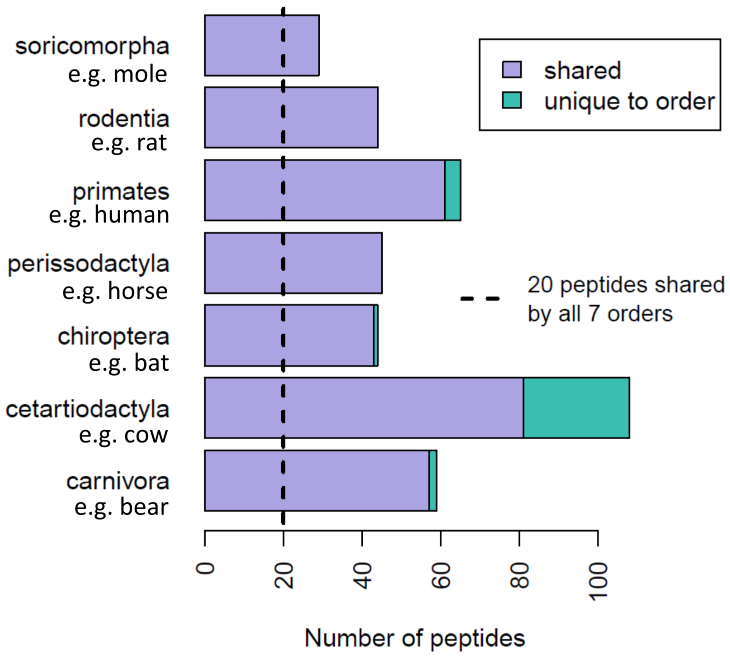

3B Number of peptides shared per order

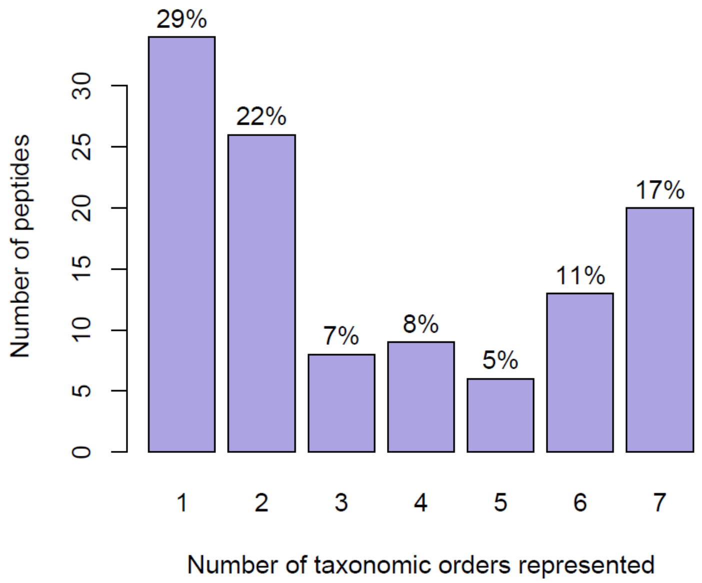

Analysis of Unique and Shared Peptides by Taxonomic Order - Tandem mass spectrometry analysis of the proteins found in the five disks identify seven mammalian orders. 3A shows the number of unique and shared peptides found in each order. 3B shows the frequency with which peptides are shared between orders.
