# Supplementary material for: Proteins, possibly human, found in World War II concentration camp artifact
Source: Sci Rep. 2022 Jul 20;12:12369. doi: 10.1038/s41598-022-16192-5 (PMC9300652; doi:10.1038/s41598-022-16192-5)
Supplement: Supplementary file 4 — Supplementary Information 4. [file 41598_2022_16192_MOESM4_ESM.pdf]

Extended Data Figure 4 Comparison of North Carolina hemoglobin peptide VNVDEVGGEALGR with ^{2}H labeled synthetic peptide.

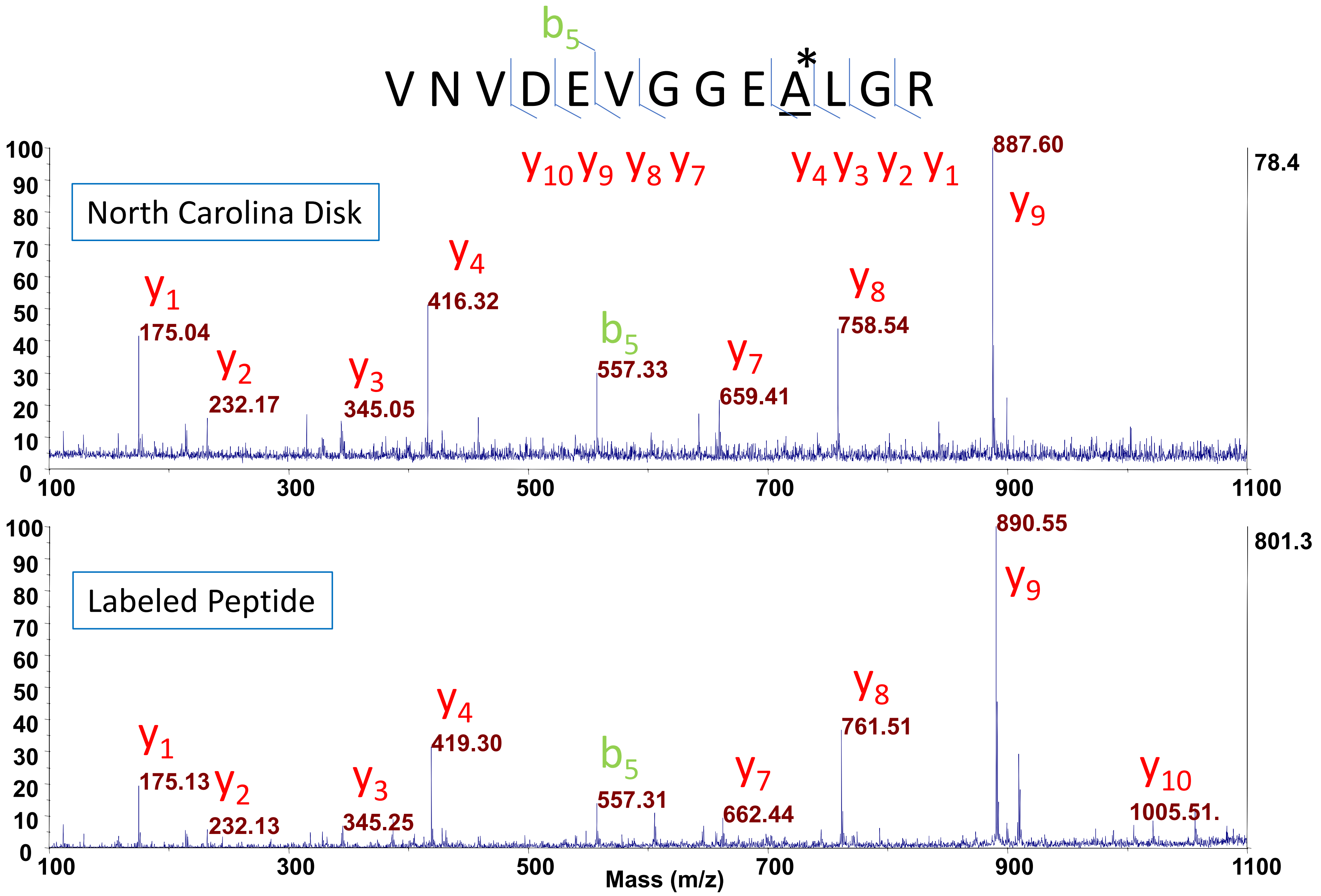

A spectral comparison between human hemoglobin peptide identified in South African disk 8002, and its synthetic counterpart with matching b and y ions. *A: ^{2}H labeled alanine
