# Supplementary material for: Proteins, possibly human, found in World War II concentration camp artifact
Source: Sci Rep. 2022 Jul 20;12:12369. doi: 10.1038/s41598-022-16192-5 (PMC9300652; doi:10.1038/s41598-022-16192-5)
Supplement: Supplementary file 5 — Supplementary Information 5. [file 41598_2022_16192_MOESM5_ESM.pdf]

Extended Data Table 1

Provenance of five disks tested by the NYC OCME.		
Disk #	Origin	Comment
None Visible	Dachau - Obtained from The Center for Holocaust, Genocide & Human Rights Education of North Carolina	Given to American solider by a former Dachau prisoner in 1945. Said to be made of human remains (*1*).
8002	Dachau - Obtained from The Cape Town Holocaust & Genocide Centre, SA	Given to a South African soldier by a former Dachau prisoner. Said to be made of human remains (*2*).
4757	Uncertain, possibly Auschwitz - Obtained from The Cape Town Holocaust & Genocide Centre, SA	No further information available.
3278	Oranienburg /Sachsenhausen - Obtained from Yad Vashem World Holocaust Remembrance Center (collection #11390)	Donated to Yad Vashem in 1985 by someone from Amsterdam who claimed it was made from ashes from a crematorium in Oranienburg/Sachsenhausen.
5910	Dachau - Obtained from Yad Vashem The World Holocaust Remembrance Center (collection #12136)	Found by an American soldier at Dachau concentration camp in 1945.
